# Ebselen Not Only Inhibits Clostridioides difficile Toxins but Displays Redox-Associated Cellular Killing

**DOI:** 10.1128/Spectrum.00448-21

**Published:** 2021-09-01

**Authors:** Ravi K. R. Marreddy, Abiola O. Olaitan, Jordan N. May, Min Dong, Julian G. Hurdle

**Affiliations:** a Center for Infectious and Inflammatory Diseases, Institute of Biosciences and Technology, Texas A&M Health Science Center, Houston, Texas, USA; b Department of Urology, Boston Children’s Hospital, Department of Surgery and Department of Microbiology, Harvard Medical School, Boston, Massachusetts, USA; University of Guelph

**Keywords:** redox stress, thiols, toxins, spores

## Abstract

Ebselen, a reactive organoselenium compound, was shown to inhibit toxins TcdA and TcdB by covalently binding to their cysteine protease domains. It was suggested that ebselen lacked antimicrobial activity against Clostridioides difficile. However, this perception conflicts with C. difficile having essential cysteine-containing enzymes that could be potential targets and the reported antimicrobial activity of ebselen against other species. Hence, we reevaluated the anti-C. difficile properties of ebselen. Susceptibility testing revealed that its activity was either slightly reduced by pyruvate found in Wilkins-Chalgren agar or obliterated by blood in brucella agar. In brain heart infusion (BHI) agar, ebselen inhibited most C. difficile strains (MICs of 2 to 8 μg/ml), except for ribotype 078 that was intrinsically resistant (MIC = 32 to 128 μg/ml). Against C. difficile R20291, at concentrations below its minimal bactericidal concentration (MBC), 16 μg/ml, ebselen inhibited production of toxins and spores. Transcriptome analysis revealed that ebselen altered redox-associated processes and cysteine metabolism and enhanced expression of Stickland proline metabolism, likely to regenerate NAD^+^ from NADH. In cellular assays, ebselen induced uptake of cysteine, depleted nonprotein thiols, and disrupted the NAD^+^/NADH ratio. Taken together, killing of C. difficile cells by ebselen occurs by a multitarget action that includes disrupting intracellular redox, which is consistent with ebselen being a reactive molecule. However, the physiological relevance of these antimicrobial actions in treating acute C. difficile infection (CDI) is likely to be undermined by host factors, such as blood, which protect C. difficile from killing by ebselen.

**IMPORTANCE** We show that ebselen kills pathogenic C. difficile by disrupting its redox homeostasis, changing the normal concentrations of NAD^+^ and NADH, which are critical for various metabolic functions in cells. However, this antimicrobial action is hampered by host components, namely, blood. Future discovery of ebselen analogues, or mechanistically similar compounds, that remain active in blood could be drug leads for CDI or probes to study C. difficile redox biology *in vivo*.

## INTRODUCTION

Clostridioides difficile, a spore-forming Gram-positive anaerobe, is the leading cause of nosocomial antibiotic-associated diarrhea (1). For about 40 years, the antibiotics metronidazole and vancomycin have been front-line treatments for C. difficile infection (CDI). However, approximately 20% or more of patients experience recurrence following therapy. The pathogenesis of C. difficile results from two glucosyltransferase toxins, toxin A (TcdA) and toxin B (TcdB), which damage the gut epithelia and are responsible for diarrheal symptoms (2). The discovery of alternative nonantibiotic CDI therapeutics has focused on blocking the action of toxins with toxin binders (e.g., tolevamer) (3), vaccines (4), antibodies such as bezlotoxumab, monoclonal antibody to TcdB (5), and microbiome-based interventions such as fecal microbiota transplantation (6, 7). Recently, the organoselenium compound ebselen [2-phenyl-1,2-benzisoselenazol-3(2H)-one] was identified from a high-throughput screen for inhibitors of the cysteine protease domains (CPDs) of C. difficile toxins (8). Ebselen inhibits both toxin A (TcdA) and toxin B (TcdB) directly by forming a covalent bond with the active site cysteine in the toxins’ CPDs. Initial studies in mice showed that ebselen attenuated CDI caused by the lab strain C. difficile 630 (8). However, in Syrian hamsters, ebselen monotherapy did not rescue these animals from CDI, but it reduced recurrence following vancomycin therapy; this was reported to be related to the ability of ebselen to promote gut microbiota recovery and to decrease inflammation (9).

Ebselen was suggested to lack antibiotic activity against C. difficile, because it targets TcdA and TcdB and did not appear to affect the bioburden in mice with CDI (8). Many other studies show that ebselen is active against Gram-positive bacteria (10–12) and yeast (13) and has multiple modes of action (13–15). Ebselen inhibits bacterial thioredoxin reductase (TrxR) by blocking electron transfer to substrates from the cofactor NADPH (14). Thioredoxin (Trx) and glutaredoxin (Grx) systems regulate various cellular functions for DNA replication (16) and protection against oxidative stress (17). Escherichia coli mutants lacking Grx but carrying the Trx system are more sensitive to ebselen, which is consistent with the compound being more active against species that contain only the TrxR system (12, 14). Our review of C. difficile genomes revealed that they possess the Trx system, appearing to lack Grx. Furthermore, C. difficile could have multiple proteins with active site cysteines (18). This prompted us to investigate what affects the antimicrobial activity of ebselen against C. difficile, in order to rationalize why it did not affect C. difficile bioburden in mice (8). Our results show that ebselen kills C. difficile but blood masks the activity of ebselen in anaerobic culture medium that is recommended for susceptibility tests. Transcriptome and metabolite analyses further demonstrated that ebselen imposed oxidative stress as part of its mechanism of action against C. difficile. Thus, ebselen is both an antibiotic and an antitoxin molecule, but its diminished activity in blood is likely to undermine its antibacterial properties *in vivo*.

## RESULTS

### Ebselen displays antimicrobial activity against C. difficile.

We first confirmed that ebselen dose-dependently protects Vero epithelial cells from TcdB cytopathy (i.e., cell rounding) (Fig. S1), with a 50% effective concentration (EC_50_) of 591 nM against TcdB from List Biological Laboratories, Inc. We then evaluated the MICs of ebselen against 12 strains of C. difficile, using the Clinical and Laboratory Standards Institute (CLSI) method with Wilkins-Chalgren agar or brucella agar (supplemented with hemin [5 mg/liter], vitamin K [1 mg/liter], and 5% [vol/vol] defibrinated sheep blood). Table 1 shows that ebselen was inactive in brucella media (MIC is ≥128 μg/ml), but in Wilkins-Chalgren agar it displayed weak to moderate MICs of 16 to 64 μg/ml, except against a ribotype 002 strain (MIC = 128 μg/ml; Table 1). When tested in BHI medium, ebselen had substantial inhibitory activity against most C. difficile strains (MICs of 2 to 16 μg/ml; Table 1); only ribotype 078 strains were intrinsically ebselen resistant (MICs of 32 to 128 μg/ml; Table S1). The difference in MICs between the types of media was investigated further. Wilkins-Chalgren agar contains hemin and sodium pyruvate, which we reasoned could alter biological redox processes in C. difficile. Hence, we supplemented BHI agars with hemin or pyruvate components. While supplementation with hemin (5 μg/ml) did not alter MICs (data not shown), the addition of 1 g/liter of pyruvate worsened ebselen MICs by 2- to 16-fold (Table 1). Next, we tested the effect of blood used in brucella agar. The activity of ebselen was ablated (MIC > 128 μg/ml; Table 1) when BHI agar was supplemented with 5% (vol/vol) defibrinated sheep blood. In contrast, the comparator vancomycin retained activity in the various media (MICs of 0.5 to 2 μg/ml) (Table 1 and Table S1). These observations revealed that ebselen not only inhibits C. difficile toxins but displays antibiotic activity against C. difficile cells.

**TABLE 1:** Antimicrobial activity of ebselen (EBS) and vancomycin (VAN) against various C. difficile strains

Strain	PCR ribotype	Agar MIC (μg/ml)^*a*^									
		BHI		BHI + blood		BHI + 1g/liter pyruvate		Brucella		Wilkins-Chalgren	
		EBS	VAN	EBS	VAN	EBS	VAN	EBS	VAN	EBS	VAN
R20291	027	4	0.5–1	>128	1–2	8	1	128	0.5–1	32	0.5–1
NR49292	001_072	4	0.5–1	>128	1–2	16	1	>128	1–2	64	0.5–1
NR49305	002	8	1	>128	1–2	16	1	>128	1–2	128	1–2
NR49294	014	4–8	0.25–0.5	>128	2–4	16	0.5	>128	2	32–64	0.25–0.5
NR49312	017	4	0.25	>128	1–2	32	1	>128	2	16	0.5
NR49323	018	4	1–2	>128	1–2	16	1	>128	2	32–64	0.5–1
NR49277	019	8	1–2	>128	1–2	16	1	>128	2	64	1
NR49300	020	4	0.5	>128	1–2	16	0.5	>128	1	32–64	0.5
NR49317	024	2–4	0.5	>128	1–2	32	1	>128	1–2	32–64	0.5
NR49314	047	4	0.25	>128	1–2	16–32	1	>128	1–2	16–32	0.5
NR49325	054	4	0.5	>128	1–2	16	1	>128	2	32–64	0.25
NR49318	106	8–16	0.5	>128	1–2	32	1	>128	2	32–64	0.5

a MICs are from three biological replicates and shown as the range, where obtained; BHI, brain heart infusion agar.

### Killing of C. difficile by ebselen.

Logarithmic cultures of R20291 (optical density at 600 nm [OD_600_] ≈ 0.2) were exposed to ebselen or vancomycin at 1 to 16 times their respective MICs to determine their MBCs (killing of 99.9% or 3 logs of cells within 24 h). The MBC of ebselen was 16 μg/ml (8 times its MIC in BHI broth of 2 μg/ml; Fig. S2); these findings were mirrored by growth curve data showing that ebselen inhibited growth at 16 μg/ml. Vancomycin killed 1.80 to 2.93 logs of bacteria at 4 to 8 μg/ml (8 to 16 times its MIC); log reductions by vancomycin are similar to those reported previously (19, 20).

### Activity of ebselen against representative gut flora.

Ebselen did not inhibit the growth of *Bacteroides* spp. and Porphyromonas uenonis (MICs ≥ 128 μg/ml; Table 2). However, it inhibited the growth of Actinomyces viscosus (MIC = 2 μg/ml), Lactobacillus crispatus (MIC = 32 μg/ml), Lactobacillus johnsonii (MICs = 8 to 16 μg/ml), Fusobacterium nucleatum (MICs = 4 μg/ml), and Fusobacterium periodonticum (MIC = 8 μg/ml). Addition of 5% (vol/vol) defibrinated sheep blood diminished the activity of ebselen. As a control, vancomycin inhibited these strains with MICs of 0.5 to 8.0 μg/ml, except against *Bacteroides* sp. HM19 (MIC of >32 μg/ml). These observations confirmed that blood impairs the antimicrobial activity of ebselen and its effect is independent of the test organism (9).

**TABLE 2:** Antimicrobial activity of ebselen (EBS) and vancomycin (VAN) against a panel of gut bacterial species

Bacteria	Strain	Agar MIC (μg/ml)^*a*^			
		BHI		BHI + blood	
		EBS	VAN	EBS	VAN
*Actinomycetes viscosus*	HM238	2	0.5	64	0.5
Bacteroides fragilis	HM20	>128	2	>128	8
Bacteroides ovatus	HM222	>128	1	>128	1
*Bacteroides sp.*	HM18	128	4	>128	8
*Bacteroides sp.*	HM19	128	>32	128	>32
*Bacteroides sp.*	HM23	128	2	>128	4
*Bacteroides sp.*	HM28	64–128	2	128	32
Lactobacillus crispatus	HM421	32	<0.25	>128	<0.25
Fusobacterium nucleatum	HM260	4	8	64	32
Fusobacterium periodonticum	HM41	8	8	128	32
Lactobacillus johnsonii	HM643	8–16	0.5	128	0.5
Porphyromonas uenonis	HM130	>128	1	>128	0.5

a MICs are from three biological replicates and shown as the range, where obtained; BHI, brain heart infusion agar.

### Transcriptome analysis of ebselen mode of action.

**FIG 1:**
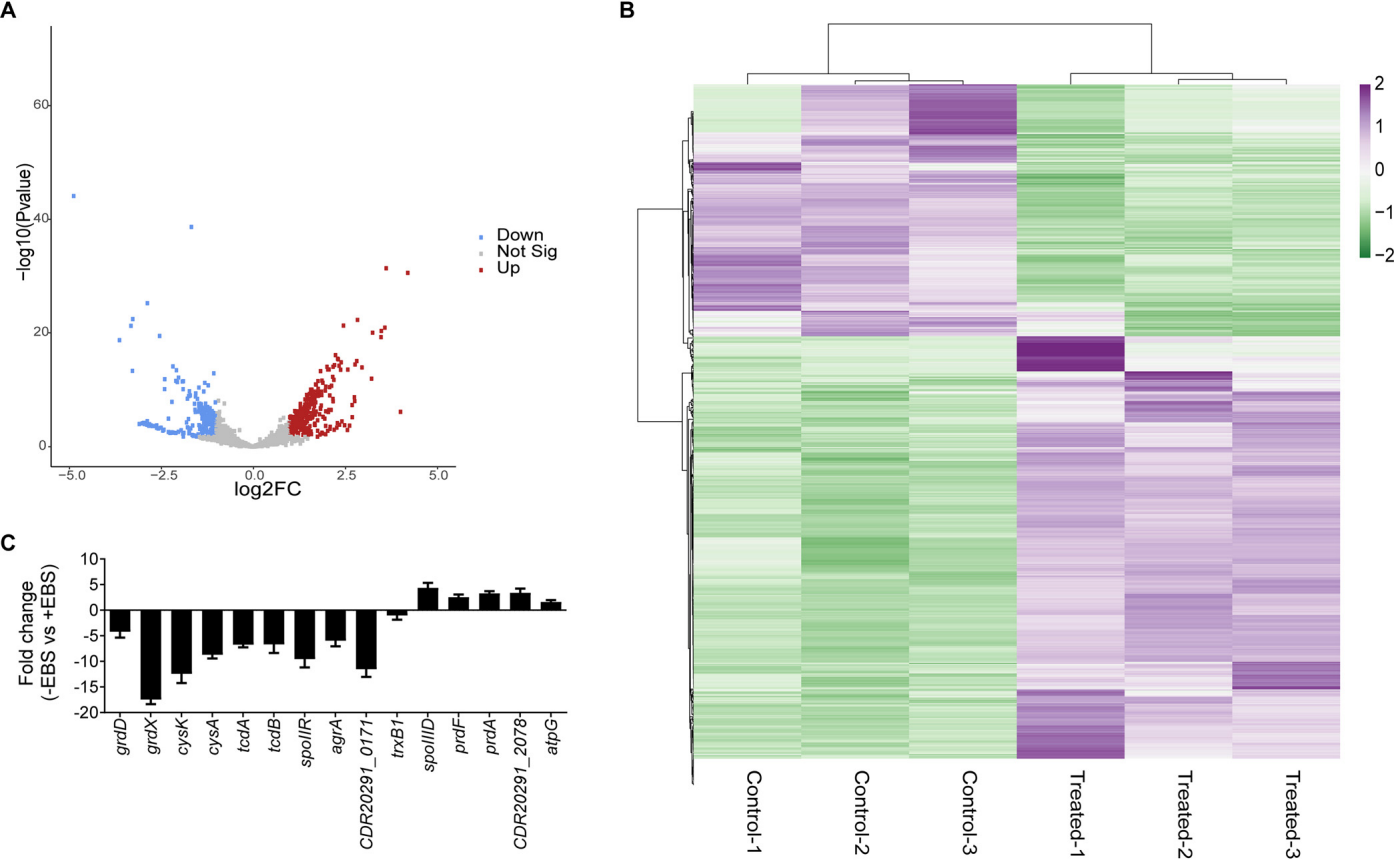
Analysis of global gene expression in the presence of ebselen. C. difficile R20291 was grown to early exponential phase (OD_600_ ≈ 0.2) and exposed to 8 μg/ml of ebselen for 30 min before RNA was extracted for sequencing. Controls were treated with dimethyl sulfoxide (DMSO). RNA-Seq data were analyzed on the Galaxy web-based platform. (A) The quality of the RNA-Seq data was analyzed by principal-component analysis, and data were visualized in volcano plots of statistical significance versus fold change. (B) Heat map of differentially expressed genes (log2FC) is shown; the color intensity provides a measure of gene expression (purple for upregulated and green for downregulated genes). The heat map was generated using Clustvis software (45). (C) mRNA levels were analyzed for various genes by RT-qPCR, and the fold change was calculated as the difference in mRNA levels of control versus ebselen-treated cells.

RNA sequencing (RNA-Seq) was adopted to assess the transcriptome response of C. difficile R20291 to ebselen at 8 μg/ml (4× MIC). The raw RNA-Seq data are deposited in NCBI database under accession number PRJNA647225 and are from three independent biological replicates. A total of 565 genes (Fig. 1A and B and Table S2) were differentially expressed by ebselen (false-discovery rate [FDR] of <0.01 and fold change of >2.0), of which 360 were upregulated and 205 were downregulated. Validation of the RNA-Seq data by reverse transcriptase quantitative PCR (RT-qPCR; using 15 upregulated and 10 downregulated genes) showed a strong correlation (Pearson’s correlation 0.85, *P* value of <0.0001; see Fig. S3). The most significantly upregulated genes belonged to the *prd* operon (*CDR20291_3098*, *CDR20291_3099*, *CDR20291_3100*, *CDR20291_3102*, *prdF*, *prdA*, and *prdB*) with changes 7- to 18-fold. In addition to these, hypothetical proteins (e.g., *CDR20291_1025*, *sbp*, *CDR20291_0571*, and *CDR20291_3388*) were also upregulated 9- to 16-fold (Table 3 and Table S2). Various ribosomal proteins and proteins involved in amino acid metabolism were upregulated. However, transcript levels for molecular chaperones were unchanged, suggesting that ebselen did not trigger protein misfolding stress. Genes encoding proteins involved in DNA replication and repair were also upregulated by more than 2-fold, consistent with ebselen being a reactive molecule. Enhanced transcription was also seen for various transporters of sugars, iron, sulfur, and amino acids. Genes involved in phosphotransferase system (PTS) carbohydrate transport (*gatA*, *gatB*, *gatC*, and *rbsR*) and the operon encoding putative phage proteins (*CDR20291_1430* to *CDR20291_1460*) were mostly downregulated with fold changes of 12- to 133-fold and 5- to 9-fold, respectively (Table 3 and Table S2). Since ebselen disrupts oxidative stress in other bacteria, we focused on validating gene signatures that suggested it imposed redox stress in C. difficile and affected sporulation and toxin production (Table 3), as discussed in below sections.

**TABLE 3:** List of selected genes^*a*^ in C. difficile R20291 that are differentially expressed by ebselen; their functional classifications are shown

Functional group/gene	Protein name	Fold change
Cysteine metabolism		
* cysA*	Serine *O*-acetyltransferase	−1.85
* cysM*	Putative *O*-acetylserine sulfhydrylase	−1.73
* CDR20291_2078*	Putative S-methylcysteine transport system	3.97
Proline reductases		
* CDR20291_3098*	d-proline reductase PrdE-like protein	18.56
* CDR20291_3099*	d-proline reductase PrdE	11.27
* CDR20291_3100*	d-proline reductase PrdD	11.22
* prdA*	d-proline reductase PrdA	7.11
* prdB*	d-proline reductase PrdB	6.88
* prdC*	Putative electron transfer protein	2.72
* prdF*	Putative proline racemase	7.83
Carbohydrate metabolism, glycolysis, and gluconeogenesis		
* gapN*	Glyceraldehyde-3-phosphate dehydrogenase	2.68
* pgK*	Phosphoglycerate kinase	2.40
* gpmI*	2,3-Bisphosphoglycerate-independent phosphoglycerate mutase	2.45
* gatC*	PTS system, galactitol-specific IIc component	−12.29
* rbsR*	Putative ribose operon repressor	−29.13
* gatB*	PTS system, galactitol-specific IIb component	−83.26
* gatA*	PTS system, galactitol-specific IIa component	−132.51
Energy generation and electron transport		
* atpD*	ATP synthase beta chain	2.21
* atpF*	ATP synthase B chain	2.07
* atpG*	ATP synthase subunit gamma	2.29
* rnfA*	Electron transport complex protein subunit A	2.20
* rnfC*	Electron transport complex protein subunit C	2.08
* rnfD*	Electron transport complex protein subunit D	3.65
* rnfE*	Electron transport complex protein subunit E	2.21
* rnfG*	Electron transport complex protein subunit G	5.10
Sporulation		
* spo0A*	Stage 0 sporulation protein A	−2.23
* spoIIAA*	Stage II sporulation protein AA	2.42
* spoIIC*	Stage II sporulation protein D	2.72
Two-component systems and transcriptional regulators associated with toxin biosynthesis		
* agrB*	Accessory gene regulator	−2.33
* agrD*	Autoinducer prepeptide	−2.23

a The above genes were selected for the following reasons. (i) Representation of most significantly upregulated (*CDR20291_3098*) and downregulated (*gatA*) genes/pathways observed from the RNA-Seq (Table S2). (ii) To validate that ebselen imposes redox stress, consistent with its mode of action in other bacteria. Genes for cysteine metabolism, proline reductases, energy generation, and electron transport led to the hypothesis that ebselen was disruptive to C. difficile redox balance. (iii) Differential expression of sporulation and *agr* genes led to the hypothesis that ebselen inhibited sporulation and toxin production. These hypotheses were substantiated in phenotypic experiments described in the main text.

### Ebselen increases cysteine pool while depleting nonprotein thiols.

Since C. difficile has three TrxR, we initially hypothesized that ebselen might inhibit one or more of these enzymes and perturb the expression of thioredoxins (*trx*) that depend on TrxR. However, ebselen did not substantially affect the transcription of any of the three thioredoxins (Table S2 and Fig. 1C). Furthermore, the MICs of ebselen were not shifted when any of the three *trx* genes (*trxA1*, *trxB1*, and *trxB3*) was expressed on plasmid pRPF185 in R20291 (Table S3). Also unaffected were transcriptions of genes regulated by the Trx system, e.g., ribonucleotide reductase, peroxiredoxin (*bcp*), and methionine sulfoxide reductase (*msrAB*). Transcription of superoxide dismutase (*sodA*) was unchanged, suggesting that ebselen did not induce the formation of reactive oxygen species (ROS). Therefore, the anti-C. difficile activity of ebselen might be independent of the Trx system. In-depth biophysical and biochemical characterization will be required to better determine whether ebselen inhibits one or more of C. difficile TrxRs.

**FIG 2:**
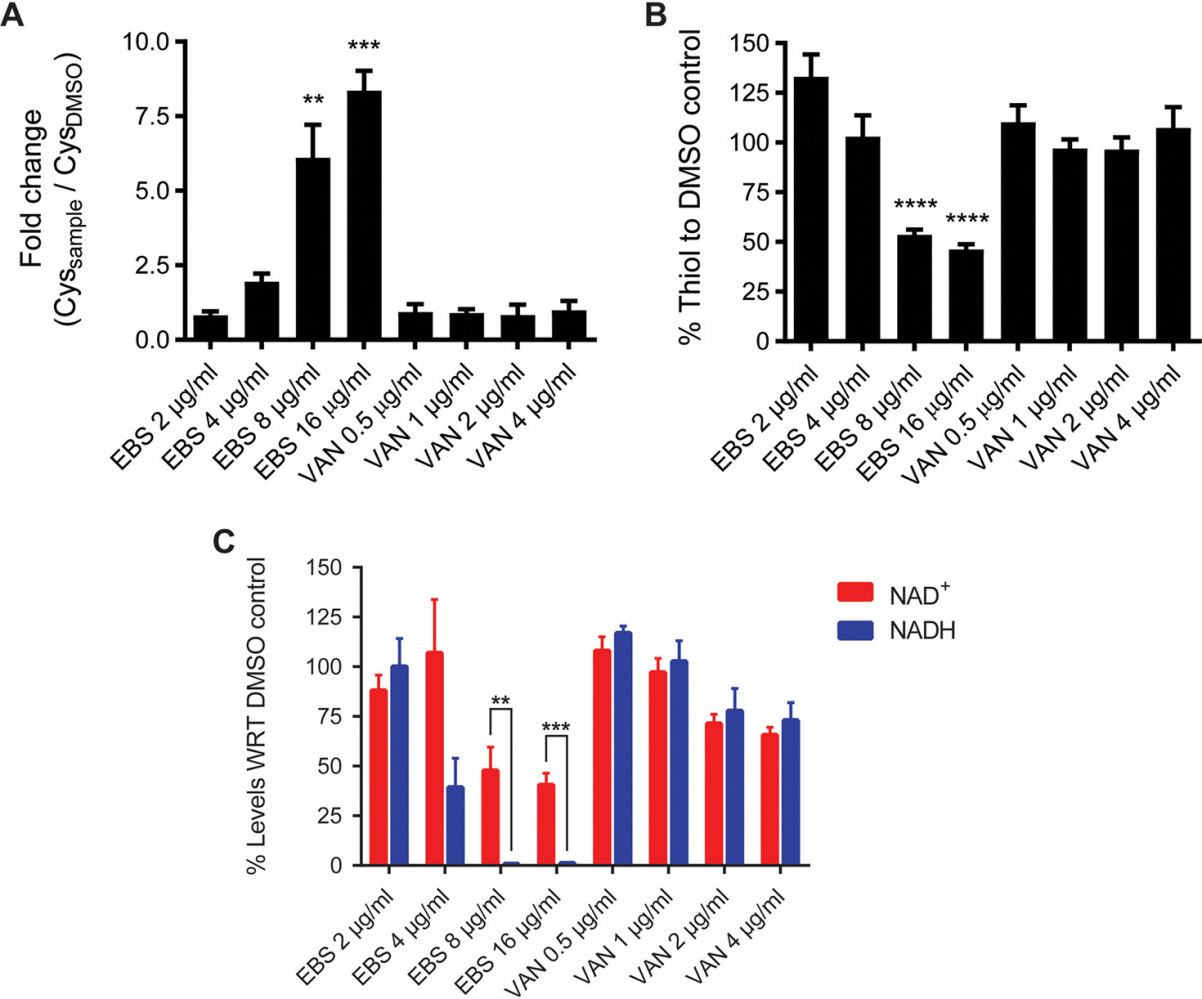
Change in cytosolic content of free cysteine, thiols, and NAD^+^/NADH in the presence of ebselen. Cultures of C. difficile R20291 were grown to early exponential phase (OD_600_ ≈ 0.4) and treated with 2, 4, 8, or 16 μg/ml of ebselen or vancomycin (0.5, 1, 2, or 4 μg/ml). The same whole-cell lysates from respective cultures were analyzed for (A) cysteine, (B) protein-free thiols, and (C) NAD^+^/NADH, using respective kits from various manufacturers. For cysteine and thiol quantifications, the fold change/percent fold change were calculated for the respective test samples relative to DMSO controls; the cysteine and thiol content in DMSO control were 203.86 ± 43.55 nmol/mg protein and 9.7 ± 3.7 μM/mg protein, respectively. For the NAD^+^/NADH plot, the relative levels of NAD+ and NADH were calculated with respect to (WRT) DMSO and the significance values were calculated for the relative percentage of NAD^+^ and NADH within the sample. The NAD^+^ and NADH content in DMSO control were 8.70 ± 1.35 and 4.06 ± 1.32 pM, respectively. The data in the plot were normalized to 1 mg of cellular protein content. A minimum of three cultures were used. Error bars indicate means ± standard error of the mean (SEM; unpaired *t* test with Welch’s correction, **, *P* < 0.01; ***, *P* < 0.001; ****, *P* < 0.0001; done using GraphPad prism version 8.4).

Our RNA-Seq data showed decreased transcription of the cysteine metabolism genes *cysM* (*O*-acetylserine sulfhydrylase) and *cysA* (serine acetyltransferase), even though their fold changes did not appear significant (1.72 and 1.84-fold, respectively) (Table S2). RT-qPCR analysis confirmed that *cysA* was downregulated by 8.7 ± 1.6-fold (Fig. 1C). Additionally, *cysK*, encoding another *O*-acetylserine sulfhydrylase, was downregulated by 12.4 ± 3.99-fold. CysM, CysA, and CysK are thought to be involved in cysteine degradation or cysteine biosynthesis from serine (21). Conversely, the expression of the ABC cystine/cysteine transporter subunit (*CDR20291_2078*) was upregulated by 3.97-fold (Table 3), which was confirmed by RT-qPCR (3.4 ± 1.6-fold upregulation) (Fig. 1C). Based on these findings, we hypothesized that ebselen might be causing the accumulation of cystine/cysteine as a source to combat its oxidative stress. To test this, we quantified intracellular concentrations of cysteine from cells exposed to various concentrations of ebselen for 1 h. Intracellular cysteine was not significantly affected by 2 and 4 μg/ml of ebselen; however, 8 and 16 μg/ml enhanced cysteine pools by 6.1 ± 2.93 (*P* = 0.0037) and 8.35 ± 1.6-fold (*P* = 0.0001), respectively (Fig. 2A). Intracellular cysteine levels were not affected by vancomycin (Fig. 2A) and metronidazole (Fig. S4). These results suggest that ebselen imposes concentration-dependent disruption of cysteine metabolism, which may correlate with its killing activities.

Low-molecular-weight (LMW) nonprotein thiols defend against reactive species (22). We quantified the free thiol content of cells to determine whether ebselen depletes LMW nonprotein thiols. The levels of LMW nonprotein thiols were not altered by 2 and 4 μg/ml of ebselen. However, significantly reduced thiol content of 46.5% ± 8.7% (*P* < 0.0001) and 53.8% ± 8.9% (*P* < 0.0001) occurred following exposure to 8 and 16 μg/ml of ebselen, respectively (Fig. 2B). When tested at the same MIC increments as ebselen, vancomycin did not affect the LMW thiol pool. Conversely, metronidazole reduced thiol content at 1 μg/ml (Fig. S4), which was observed previously (22). Based on these observations, we speculate that C. difficile adopts LMW thiols to detoxify ebselen. It is also plausible that cysteine is being accumulated to replete the thiol pool. It may also serve as an amino acid electron donor in the Stickland pathway, which was activated by ebselen (Table 3). This pathway couples the oxidation of one amino acid (e.g., cysteine) to the reduction of another (e.g., proline) (23, 24).

### Regulation of NAD^+^-generating pathways and energy generation.

**FIG 3:**
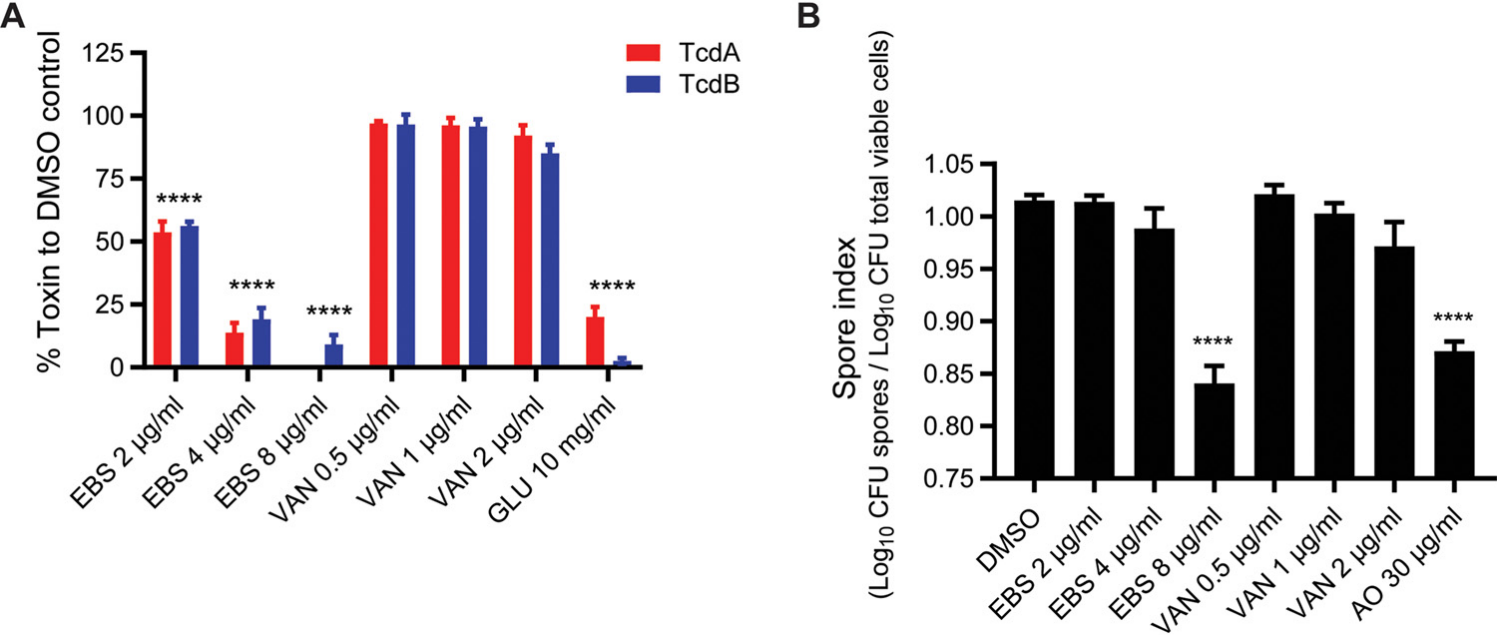
Effects of virulence by growth inhibitory concentrations of ebselen. C. difficile R20291 was grown to early exponential phase (OD_600_ ≈ 0.2) and treated with 2, 4, or 8 μg/ml of ebselen (EBS). (A) After exposure for 24 h, both TcdA (red bars) and TcdB (blue bars) were measured from culture supernatants by ELISA. Vancomycin (0.5, 1, 2, or 4 μg/ml) and glucose (1% wt/vol) were used as controls. Data obtained from four biological replicates were compared with respective DMSO controls. (B) Sporulation was analyzed after 5 days, and the spore index expressed log_10_ spores to log_10_ total viable population. Vancomycin (VAN) at 0.5, 1, and 2 μg/ml and acridine orange (AO) at 30 μg/ml was used as the control. Error bars indicate means ± SEM (unpaired *t test w*ith Welch’s correction, ****, *P* < 0.0001; done using GraphPad prism version 8.4).

C. difficile proline reductase is a selenoenzyme that helps maintain cellular redox balance and generate energy via the Stickland pathway (23). Proline reductase is thought to be the preferred route for NAD^+^ regeneration in C. difficile (25). Similar to proline reductases, glycine reductase also regenerates NAD^+^ from NADH in the alternate branch of the reductive pathway (25). The most significantly induced genes by ebselen belonged to the proline reductase operon (Table 3); RT-qPCR confirmed that *prdA* and *prdF* were upregulated by 3.3 ± 1.1- and 2.5 ± 1.2-fold, respectively (Fig. 1C). In contrast, RT-qPCR showed that the glycine reductase subunits (*grdD* and *grdX*) were downregulated by 4.2 ± 1.99- and 17.4 ± 2-fold, respectively (Fig. 1C); *grdD* and *grdX* were not significantly downregulated in the RNA-Seq (Table S2). Upregulation of proline reductase genes, and downregulation of glycine reductase genes, suggested that proline metabolism was activated by ebselen to regenerate NAD^+^. We therefore quantified the cellular ratio of NAD^+^/NADH in ebselen-exposed cells. As shown in Fig. 3C, ebselen, in a dose-dependent fashion, decreased the cellular ratio of NAD^+^/NADH. As expected, vancomycin did not perturb the NAD^+^/NADH ratio. Proline reduction is thought to also result in a proton motive force by coordinating with the electron transport Rnf complex, and the resulting ion gradient may be used to generate ATP via the ATP synthase (26). Consistent with upregulation of proline reductase genes, in the RNA-Seq, ATP synthase subunits *atpD*, *atpF*, and *atpG* were upregulated, along with the electron transport protein complex encoded by *rnfA*, *rnfC*, *rnfD*, *rnfE*, and *rnfG* (Table 3). RT-qPCR showed that *atpG* was upregulated by 1.62 ± 0.6-fold (Fig. 2C). Hence, in ebselen-exposed C. difficile, it is plausible that proline may be metabolized to generate energy and NAD^+^; furthermore, cysteine might be serving as an electron donor amino acid.

Table 1 shows that supplementation of BHI with 1 g/liter of pyruvate weakened the activity of ebselen against C. difficile. Pyruvate enters various metabolic pathways in C. difficile. However, we did not observe a significant expression of pyruvate-ferredoxin oxidoreductase (PFOR; *nifJ*, 1.68-fold) and pyruvate lyase (*plfB*, 1.78-fold). Hence, it is not entirely clear which pathway pyruvate is being metabolized to affect the action of ebselen.

### Ebselen inhibits toxin production in C. difficile.

Various factors influence the expression of *tcdA* and *tcdB* (27). For example, activation of PrdR represses *tcdA* and *tcdB* (28), while Agr, a two-component regulatory system, enhances *tcdA* and *tcdB* expression (29). The *agr* genes *agrB* and *agrD*, encoding the precursor for the quorum-sensing signal, were downregulated by 2.33- and 2.23-fold, respectively, by ebselen (Table 3). Confirmation by RT-qPCR showed that *agrA* expression was decreased by 5.9 ± 2.4-fold (Fig. 2C). These observations implied that ebselen could reduce toxin production through PrdR activation and downregulation of *agr* genes. RT-qPCR showed that *tcdA* and *tcdB* were downregulated 6.74 ± 1.14- and 6.68 ± 3.76-fold, respectively (Fig. 1C). We next quantified toxins by enzyme-linked immunosorbent assay (ELISA) in cultures that were exposed to various concentrations of ebselen and other test compounds for 24 h. As shown in Fig. 3A, ebselen (2 μg/ml, i.e., MIC) inhibited production of both TcdA and TcdB by ∼45% (*P* < 0.0001), while 4 and 8 μg/ml of the compound caused >80% inhibition (*P* < 0.0001). As a positive control, glucose (1% wt/vol) strongly inhibited the production of both TcdA (∼80%, *P* < 0.0001) and TcdB (97%, *P* < 0.0001) (Fig. 3A). In contrast, vancomycin did not influence toxin production at up to 4 times its MIC, which is consistent with several reports (19, 30, 31).

### Ebselen inhibits C. difficile spore production.

Analysis of the RNA-Seq data revealed a mixed transcriptional response for sporulation genes (Table 3). While mRNA for stage 0 (*spo0A*) was significantly downregulated (2.23-fold), the stage II genes (*spoIIAA* and *spoIIC*) were upregulated (2.42- and 2.72-fold, respectively). This suggested that ebselen affected the initiation of sporulation. Spore production was therefore assessed by exposing cultures to various ebselen concentrations for 5 days. This revealed that sporulation was inhibited only at 8 μg/ml (4-fold MIC; Fig. 3B), since 10.67% ± 6.74% of spores were present in the total population after treatment (based on CFU/ml values in Fig. S5), which was comparable to the positive control acridine orange (30 μg/ml). However, over the 5 days, ebselen (8 μg/ml) also caused a 0.77 to 2.47 log reduction in viable cells, implying that spore reduction was coupled to cell death. Changes in total viable counts and spores are shown in Fig. S5.

## DISCUSSION

Our findings lead to an updated model of ebselen anti-C. difficile properties, whereby it protects epithelial cells from C. difficile toxins, kills C. difficile cells, and blocks the biogenesis of toxins and spores. The polypharmacological action of ebselen against C. difficile is somewhat similar to observations in Staphylococcus aureus, where it inhibits the synthesis of α-hemolysin, protein, DNA, RNA, and cell envelope (10). Interestingly, we could not select stable ebselen-resistant mutants in R20291 (data not shown). However, the occurrence of ebselen-resistant ribotype 078 suggests that ebselen’s antibacterial activity could be overcome by C. difficile. Ribotype 078 (clade 5, ST11), which is common in farm animals, has vastly diverged from other clinically occurring clades (32, 33). It appears to be adept at acquiring mobile elements, such as a unique, large, >100 kb transposon Tn*6164* that contains nonclostridial genes. However, it is not known how ribotype 078 strains exhibit resistance to ebselen. In addition to antimicrobial resistance, there is a potential for host factors, such as blood components, to affect the antibacterial efficacy of ebselen *in vivo*, which would make it unsuitable for CDI. Indeed, ebselen reacts with thiols of the plasma protein albumin and glutathione that is found in blood (34, 35). This would reduce the free fraction of unreacted ebselen for cellular activity against C. difficile. Because the 50% inhibitory concentration (IC_50_) of ebselen against TcdB is at least 10-fold lower than its MIC against C. difficile, it is likely that the antibacterial activity will be more disrupted than its antitoxin activity. Of note, frank blood is uncommon in CDI, but patients with underlying inflammatory bowel disease are prone to intestinal bleeding (36, 37). It is plausible that inactivation of ebselen by host factors could account for reported inability of ebselen to disrupt the microbiota of mice (9). Nonetheless, studies will be required to determine how the efficacy of ebselen is affected by blood and thiol-containing host factors.

Our mechanistic studies suggest that oxidative stress caused by ebselen leads to depletion of LMW thiols, a redox imbalance of NAD^+^/NADH, and intracellular uptake of cysteine. It is likely that the cellular action of ebselen differs from the reactive prodrug metronidazole. For example, metronidazole does not induce the production of proline reductase, as shown from proteome analyses (38, 39). Conversely, our observed increase in NAD^+^ in ebselen-exposed cells correlated with upregulation of the proline reductase operon. This likely reflects the major role of proline reductase in regenerating NAD^+^ from NADH (25). Toxin biosynthesis was also affected substantially by ebselen at its MIC. This could be the result of activation of the proline reductase operon, repression of the *agr* genes, or intracellular uptake of cysteine, which are all expected to reduce toxin biosynthesis (27, 29). Currently, there is growing momentum to better understand biological redox processes in C. difficile metabolism (26). We suggest that ebselen could be useful to probe hierarchical pathophysiological changes that occur in response to oxidative stress. From a drug discovery standpoint, future analogs of ebselen or similarly reactive molecules will need to consider the potential for *in vivo* drug efficacy to be hindered by thiol-containing host components, such as blood.

## MATERIALS AND METHODS

### Bacterial strains, growth conditions, and antibiotics.

Except for C. difficile R20291, the various PCR ribotypes of C. difficile and human gut bacteria were from Biodefense and Emerging Infectious Research Resource Repository (BEI Resources, Manassas, VA) and the American Type Culture Collection (ATCC, Manassas, VA). C. difficile strains were grown in brain heart infusion (BHI) agar or broth medium at 37°C in an anaerobic chamber (Don Whitley A35 anaerobic chamber). Other species were routinely grown in brucella agar supplemented with 5% (vol/vol) defibrinated sheep blood (Hardy Diagnostics), 5 mg/liter hemin, and 10 mg/liter vitamin K1. The antibiotics were purchased from Sigma (vancomycin), Acros organics (metronidazole), and Enzo life sciences (ebselen).

### Susceptibility testing.

MICs were determined by the agar dilution method, as described previously (40). Briefly, serially diluted test compounds were added to molten agars of respective media, and 3 μl (∼10^5^ CFU/ml) of overnight cultures were spotted onto agars using the semiautomated liquid handling benchtop pipettor (Sorenson Bioscience Inc.). After anaerobic incubation at 37°C for 24 h, the lowest concentration of compound inhibiting visible growth was recorded as the MIC. Agars used were BHI, brucella with above supplementation, and Wilkins-Chalgren. When needed, BHI was supplemented with 5% (vol/vol) defibrinated sheep blood, 1 g/liter Na-pyruvate, or hemin (5 mg/liter).

### MBC determination.

MBCs were determined by the agar plating method, as described previously (41). Cultures were grown to OD_600_ of ∼0.2 and exposed to various concentrations of ebselen. Total viable counts were determined, at time zero and 24 h, by plating serial dilutions of bacteria onto BHI agar plates. The MBC was defined as the lowest concentration of compound that kills 3 logs of cells from time zero.

### Effects on growth.

The effects of ebselen and vancomycin on the growth of C. difficile were evaluated by growth kinetics in 96-well microtiter plates in 250 μl. Overnight cultures were diluted 1:100 in fresh growth medium and grown until an OD_600_ of ∼0.2. Approximately 125 μl of respective cultures was added to an equal volume of medium containing compounds at various concentrations. Automated growth was recorded every 30 min for 24 h with shaking before each read in the Synergy H1 microplate reader (BioTek Instruments), enclosed in an anaerobic chamber (Coy Laboratory Products).

### Transcriptome analysis.

Overnight cultures of C. difficile R20291 were inoculated in 50 ml of fresh BHI medium and cultured until an OD_600_ of ∼0.2 (*T*_0_). Ebselen was added to a final concentration of 8 μg/ml (4-fold MIC) and cultures were incubated for a further 30 min; corresponding cultures that were not exposed to ebselen served as controls. Cells were harvested after the addition of one volume of RNAprotect bacterial reagent (Qiagen) and centrifugation at 4,000 × *g* for 10 min. Cell pellets were resuspended in 700 μl of Qiazol lysis reagent (Qiagen) and lysed in a FastPrep cell disruptor at force 50 for 5 min. The total RNA was extracted using the RNeasy minikit (Qiagen) according to the manufacturer’s instructions. RNA Sample QC, DNase treatment, library preparations, and HiSeq 2 × 150 paired-end sequencing were performed by GENEWIZ (South Plainfield, NJ, USA).

### Bioinformatic analysis.

Raw FASTQ files were uploaded onto the Galaxy platform (https://usegalaxy.org/). Quality control and trimming were done using FastQC (Babraham Bioinformatics) and Trim Galore, respectively. Reads were mapped to R20291 as a reference and were performed using BWA-MEM program. Counts per read were generated with HTSeq-count, and count matrix was generated with Column Join tool program. The count matrix file was uploaded onto Degust (http://degust.erc.monash.edu/) to generate differential gene expression file using edgeR (cutoffs, |fold change| [FC] of 2.0 and FDR of 0.01).

### Gene expression analysis by reverse transcriptase quantitative PCR.

RNA extraction was performed as indicated above. cDNA was prepared from 10 μg of total RNA using qScript cDNA supermix (Quanta Biosciences). Quantitative PCR was performed with qScript SYBR green RT-qPCR master mix (Quanta Biosciences) using Applied Biosystems ViiA7 RT-PCR system. The threshold cycle (*C_T_*) values obtained were normalized to the housekeeping 16S rRNA, and the results were calculated using the 2^ΔΔC^_T_ method (42). The primers used are shown in Table S4.

### Cysteine quantification.

Overnight cultures of C. difficile R20291 were diluted 20-fold into a fresh medium and grown until an OD_600_ of ∼0.3. Compounds were added at various concentrations and cultures were incubated for 1 h. Cells were harvested by centrifugation (4,000 × *g* for 10 min) and cell pellets were resuspended in ice-cold phosphate buffer. Cells were lysed in FastPrep (Qiagen) for 10 min and centrifuged at 21,100 × *g* for 5 min, and cysteine content was quantified using the cysteine assay kit from Sigma-Aldrich according to the manufacturer’s instructions. Cellular cysteine levels were normalized in cell lysates by protein content determined using Pierce BCA protein assay kit.

### Thiol quantification.

The thiol quantification was performed as described previously (22). Briefly, samples of the cell lysates used above were treated with 5% (wt/vol) trichloroacetic acid for 15 min at room temperature. Precipitated proteins were removed by centrifugation (21,100 × *g* for 5 min), and the pH of supernatants was neutralized with 1 M Tris-base. LMW thiols were quantified using thiol fluorescent detection kit (Invitrogen) according to the manufacturer’s instructions and were normalized by protein content.

### NAD/NADH quantification.

Cells were harvested as above, resuspended in phosphate-buffered saline (PBS) buffer, and diluted with an equal volume of base solution (0.2 N NaOH with 1% [wt/vol] dodecyl trimethylammonium bromide [DTAB]). After lysing in FastPrep, lysates were centrifuged at 21,100 × *g* for 5 min. Both NAD^+^ and NADH were quantified using NAD/NADH-GLO kit (Promega) according to the manufacturer’s instructions. NAD^+^ and NADH concentrations were normalized by protein content.

### Toxin quantification.

TcdA and TcdB were quantified from supernatants of 24-h cultures of R20291, using the C. difficile toxin A or B ELISA kit (tgcBIOMICS), according to the manufacturer’s instructions. Cultures were exposed to ebselen, vancomycin, or glucose for 24 h.

### Sporulation assay.

This assay was performed as described previously (19). Briefly, cultures were grown to an OD_600_ of ∼0.3 in BHI before the addition of test compounds at various concentrations. Cultures were incubated for 5 days, and sporulation was evaluated as the ratio of heat-resistant spores per total viable count.

### Cell-rounding assay.

This assay was used to measure toxin activity, as C. difficile toxins cause cell rounding of tissue cultures, and was performed as described previously (43) with slight modifications. Briefly, Vero cells (monkey kidney epithelial cells) from ATCC were aliquoted in 24-well plates at a density of 10^3^ cells/ml and incubated overnight at 37°C in a CO_2_ incubator. Cells were treated with serially diluted concentrations of ebselen (ranging from 125 ng/ml to 128 μg/ml) for 30 min, followed by the addition of TcdB (List Biologicals Laboratories, Inc.) to a final concentration of 20 ng/ml. The plates were incubated at 37°C in a CO_2_ incubator for 2 h and then observed with phase-contrast microscope (Nikon Diaphot 200/300 inverted microscope) with counting from 5 views per well.

### Expression of thioredoxin genes in C. difficile.

Three thioredoxin homologs (*trxA1*, *trxB1*, and *trxB3*) were each cloned and expressed in strain R20291 using the vector pRPF185 (44). The cloning primers are listed in Table S4; PCR amplicons of the genes with their own ribosome-binding site (RBS) were cloned into SacI and BamHI sites of pRPF185. Successful clones were confirmed by PCR and restriction analysis. Genes were expressed from the pTet promoter following induction with 0.25 μg/ml of anhydrotetracycline.

### Data availability.

RNA-Seq data are deposited in NCBI database under accession number PRJNA647225.
