# Supplementary material for: Ebselen Not Only Inhibits Clostridioides difficile Toxins but Displays Redox-Associated Cellular Killing
Source: Microbiol Spectr. 2021 Sep 1;9(2):e00448-21. doi: 10.1128/Spectrum.00448-21 (PMC8557875; doi:10.1128/Spectrum.00448-21)
Supplement: SUPPLEMENTAL FILE 2 — Supplemental material. Download SPECTRUM00448-21_Supp_2_seq1.pdf, PDF file, 0.4 MB. [file spectrum00448-21_supp_2_seq1.pdf]

SUPPLEMENTARY INFORMATION

Ebselen Not Only Inhibits *Clostridioides difficile* Toxins, But Displays Redox Associated Cellular Killing

Ravi K.R. Marreddy[1], Abiola O. Olaitan[1], Jordan N. May[1], Min Dong[2], Julian G. Hurdle[1]*

[1]Center for Infectious and Inflammatory Diseases, Institute of Biosciences and Technology, Texas A&M Health Science Center, 2121 West Holcombe Blvd, Houston, Texas 77030, USA

[2]Department of Urology, Boston Children's Hospital, Department of Surgery and Department of Microbiology, Harvard Medical School, Boston, Massachusetts 02115, USA

*Correspondence and requests for materials should be addressed to J.G.H (jhurdle@tamu.edu).

Table of contents

Title	Page #
Supplementary results[a]	S3
**Figure S1:** Ebselen protects Vero cells from TcdB	S3
**Figure S2:** Effect of ebselen on bacterial growth and survival	S4
**Figure S3:** Pearson's correlation of RNA-seq and RT-qPCR results	S5
**Figure S4:** Change in cytosolic content of free cysteine, thiols and NAD^+/ NADH in presence of metronidazole	S6
**Figure S5:** Total viable and spore counts under various conditions.	S7
**Table S1:** Antimicrobial activity of ebselen and vancomycin against various *C. difficile* ribotype 078 strains	S7
**Table S3:** Minimal Inhibitory Concentrations for ebselen and vancomycin against *C. difficile* R20291 overexpressing thioredoxin genes	S8
**Table S4:** Primers used in this study	S9

[a]Table S2 concerning list of all significantly expressed genes can be found as a seperate excel sheet.

SUPPLEMENTARY RESULTS

Figure S1

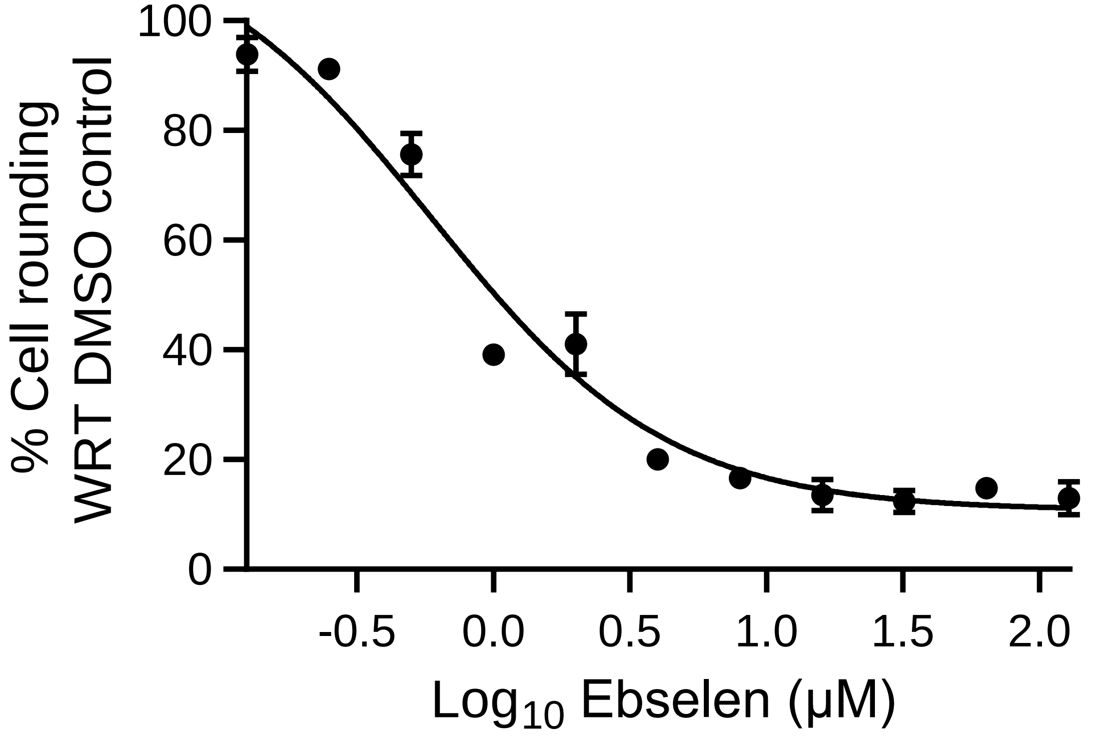

Figure S1: Ebselen protects Vero cells from TcdB. This assay was performed in 24-well plate in ~10^3 cells/ml of Vero (African monkey kidney epithelial) cells were treated with various concentrations of ebselen for 30 min. Ebselen treated cells were challenged with 20 ng/ml of TcdB for 2 h followed by phase contrast microscopy to measure cytotoxic effects. Data from four biological replicates were compared with their respective DMSO controls. The EC_{50} was 591 nM using Graphpad prism version 8.4.

Figure S2

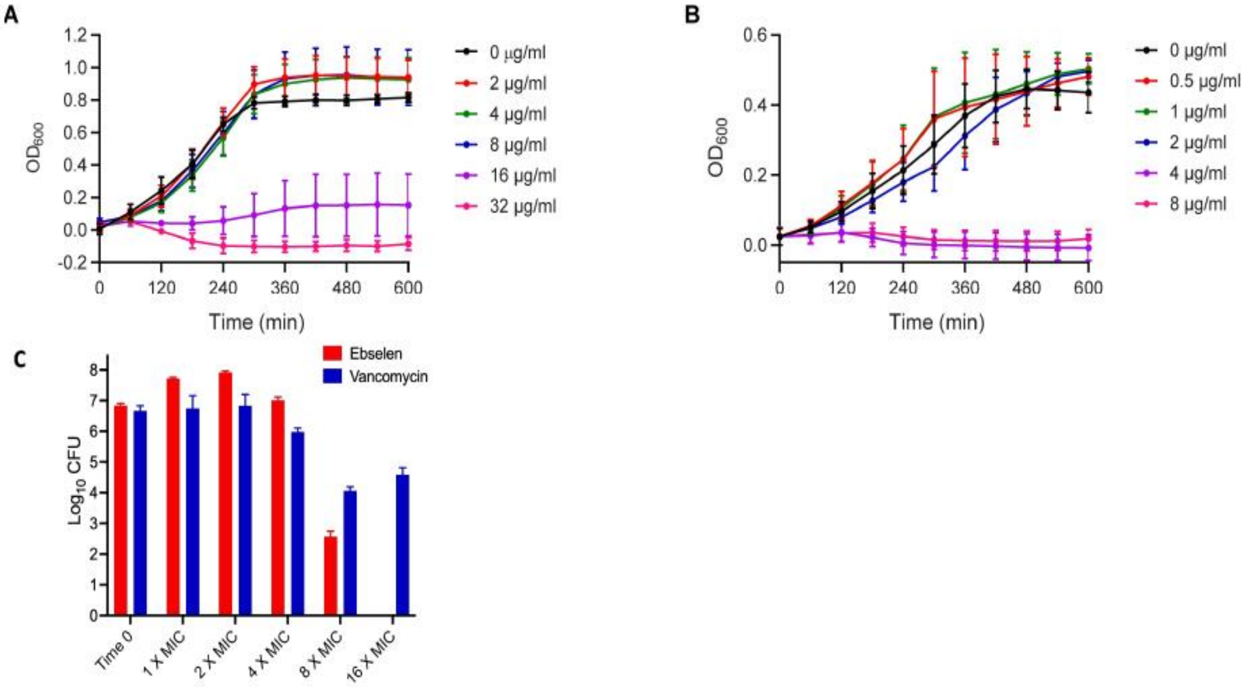

Figure S2: Effect of ebselen on bacterial growth and survival of *C. difficile* R20291 in Brain heart infusion broth. A) effect of ebselen on growth; **B)** effect of vancomycin on growth; and **C)** effect of ebselen and vancomycin on bacterial viability. *C. difficile* R20291 was grown to exponential phase (OD_{600} 0.2-0.3) and treated with indicated concentrations of ebselen or vancomycin. The MIC of ebselen is 2 µg/ml and vancomycin 0.5 µg/ml. **Growth kinetics** were performed in 96-well microtiter plates with total volume of 250 µl. **MBCs** were determined by exposing cells to the antibiotics at indicated concentrations for 24 h and viable counts at time 0 and 24 h compared. There were no colonies from cultures treated with ebselen at 16 x MIC. MBC is 8 x MIC, representing killing of 3 logs of bacteria relative to time 0. Error bars indicate means ± SEM from minimum of three independent experiments.

Figure S3

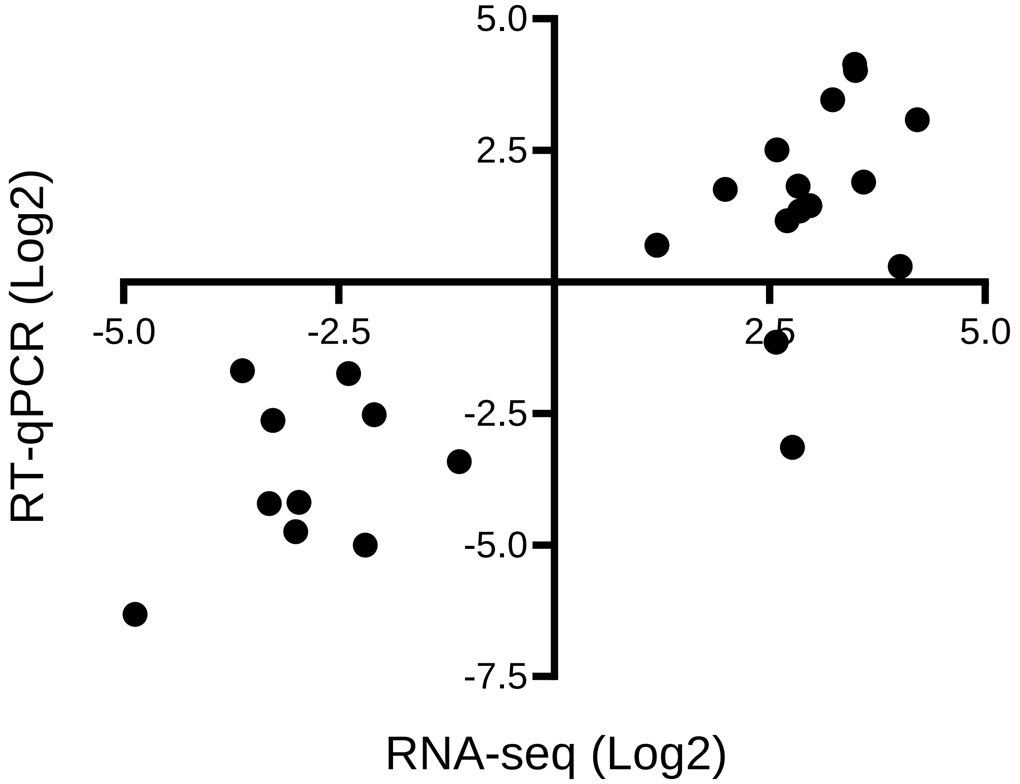

Figure S3: Confirmation of the RNA-seq results with RT-qPCR. A panel of 25 differentially expressed genes (15 – upregulated and 10 – downregulated) representing a wide range of expression fold-change in RNA-seq were further validated by RT-qPCR. The fold change (mean ± SEM) was calculated as the difference in mRNA levels of control vs ebselen treated cells. The scattered plot represents the correlation found for Log2 fold-change in expression levels determined by RNA-seq vs RT-qPCR. The Pearson correlation coefficient (0.8517; $p < 0.0001$) demonstrates a high degree of correlation between the RNA-seq and RT-qPCR.

Figure S4

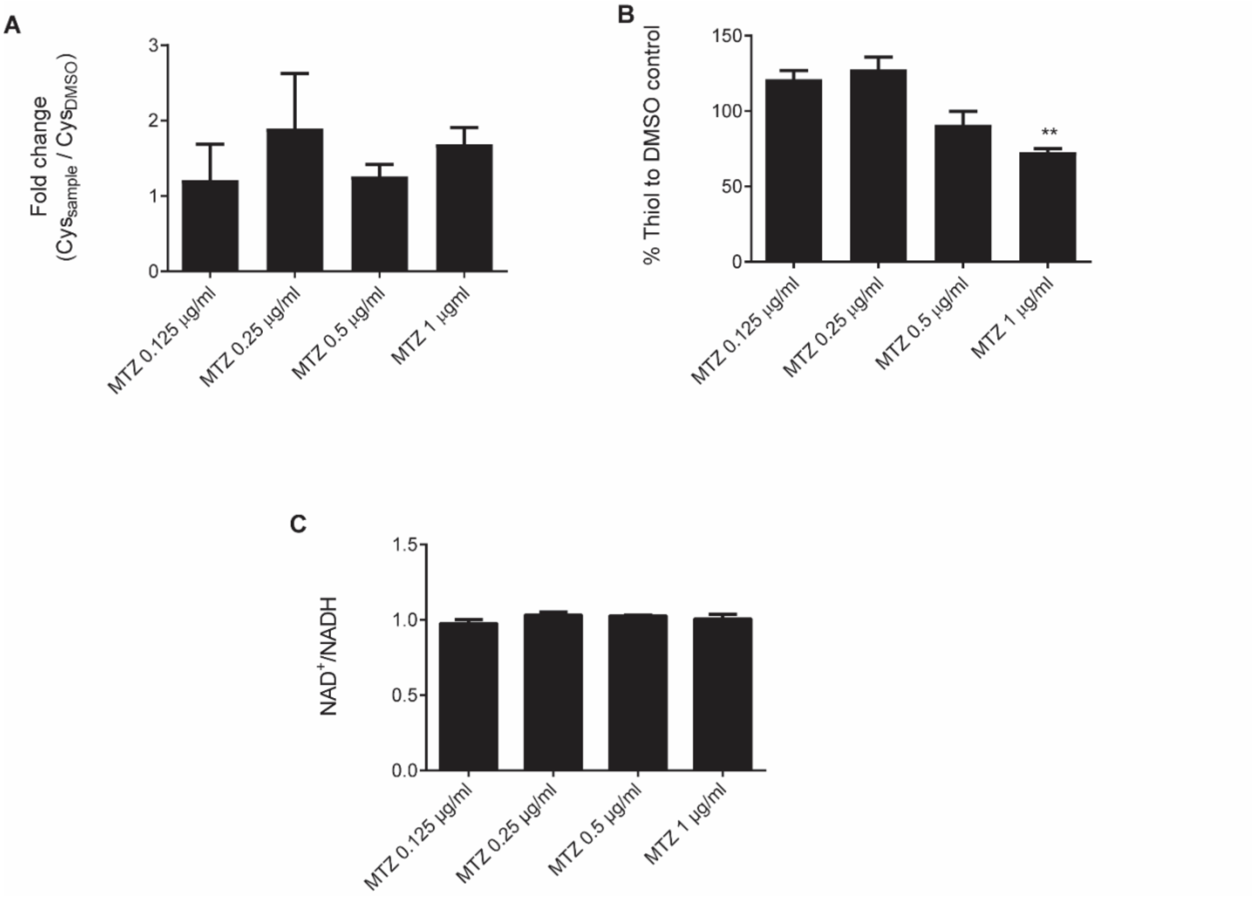

Figure S4: Change in cytosolic content of free cysteine, thiols and NAD^+/ NADH in presence of metronidazole (MTZ). Cultures of *C. difficile* R20291 were grown to early exponential phase (OD_{600} ≈ 0.4) and treated with 0.125, 0.25, 0.5 or 1 µg/ml of metronidazole. Whole cell lysates were analyze for: **(A)** cysteine **(B)** protein free thiols and **(C)** NAD^+/NADH, using respective kits from various manufacturers. The data is representative of two biological and two technical replicates. For technical replicates, each culture was split into two parts and processed independently. Error bars indicate

means ± SEM (unpaired t-test with Welch's correction **$p<0.01$; done using Graphpad prism version 8.4).

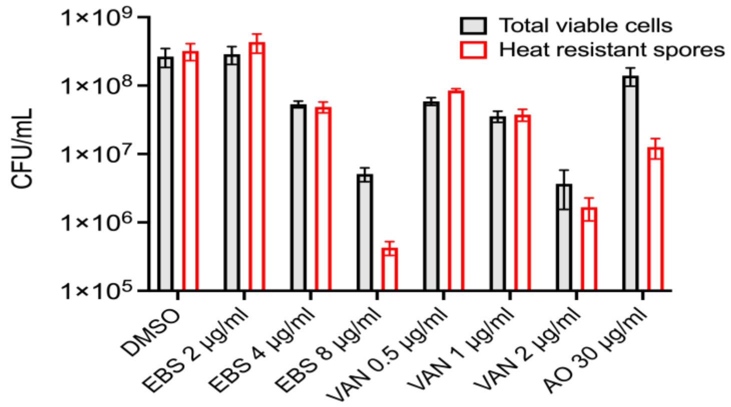

Figure S5: Total viable and spore counts over 5 days are shown. This data was used to calculate spore indices in figure 3B of the main text. Error bars indicate means ± SEM. CFU = colony forming units.

Table S1. Antimicrobial activity of ebselen (EBS) and vancomycin (VAN) against various *C. difficile* ribotype 078 strains.

Strain	PCR-ribotype	Agar MIC (µg/ml)[A]	
		EBS	VAN
NR49310	078	128	0.5
NR49311	078	64-128	0.5
MT2748	078	64-128	0.5
SC69	078	64-128	0.5
MT3245	078	128	0.25
MT3067	078	32	0.5
MT3644	078	64-128	0.5
MT2694	078	64-128	0.5
MT2814	078	64-128	0.5
MT2705	078	128	0.5

[A]MICs are from three biological replicates and shown as the range, where obtained. MIC tests were performed with brain heart infusion agar.

Table S3. Minimal inhibitory concentrations of ebselen (EBS) and vancomycin (VAN) against *C. difficile* R20291 overexpressing thioredoxin genes.

**Strain**	**Agar MIC's (µg/ml)[A]**			
	**EBS**		**VAN**	
	ATc (0 ng/ml)	ATc (100 ng/ml)	ATc (0 ng/ml)	ATc (100 ng/ml)
C. difficile R20291 pRPF185	4	4 – 8	1	0.5 – 1
C. difficile R20291 pRPF185-*trxB1*	4	4 – 8	1	0.5 – 1
C. difficile R20291 pRPF185-*trxB2*	4	8	0.5 – 1	0.5
C. difficile R20291 pRPF185-*trxB3*	4	4 – 8	0.5 – 1	0.5

[A]MICs are from four biological replicates and are shown as the range, where obtained. MICs were performed with brain heart infusion agar. Gene expression was induced by supplementing the medium with 100 ng/ml of anhydrotetracycline (ATc).

Table S4. Primers used in this study.

No	Name	Sequence
Cloning primers		
1	trxB1_F	ATGCACGAGCTCTCATATTTATAAAAAATGGGTGAGTATTATGAG
2	trxB1_R	ATGCACGGATCCTTAGTGATGGTGATGGTGATGAGCCCTCTTTAATTTATCTAAATAGTAAAC
3	trxB2_F	ATGCACGAGCTCAAGAGAGGTAAACGTTATGGTAGATATCATT
4	trxB2_R	ATGCACGGATCCTTAGTGATGGTGATGGTGATGTTCAACATTTATATAATTAGCTGCTTGA
5	trxB3_F	ATGCACGAGCTCTTGGGGAGGATAAGTCAAAATGG
6	trxB3_R	ATGCACGGATCCTTAATGGTGATGGTGGTGGTGGTTCCCAAATTTTTCATCAATGTATTTC
RT-qPCR primers		
7	16S_F	GGGAGACTTGAGTGCAGGAG
8	16S_R	GTGCCTCAGCGTCAGTTACAGT
9	grdX_F	GGCAGAGAAGTTTTGTTTAAAGACACAAC
10	grdX-R	AACGATTTCCTTTCTTTAGTATAACAGTCC
11	grdD_F	GTTACTACACTTGGAAGTGAACATGGTG
12	grdD_R	AATTCTTCCATTTTTGCATGCATTTC
13	cysK_F	ATTAGTTCCAGGTCAAGCAGTTGTGG
14	cysK_R	AGGCATTTGGTCTACAAGAACTACTTC
15	cysA_F	GATCCAGCAGCTAGGTCTAAGATAGAAG
16	cysA-R	CCATTTTAGCACCAGGATGAATTTC
17	tcdA_F	AGACCGTTGGGAAATGATAAAATTAG
18	tcdA_R	GATTTTTCACTTTTACTTTCTATAATGAG
19	tcdB_F	CTCATCACTTGGCGATATGG
20	tcdB_R	TTATATCTGTTCTCGATTTGTTTTAC
21	spoIIR_F	CAGCAATACAGATGAAGATCAAG
22	spoIIR_R	CTTATTTCTTCCAGACTACTATATTCC
23	agrA_F	AAGGATGTTTAAGAATTGTGATTAGTATAGG
24	agrA_R	ACTCTTCTCCTGATGAAAATTCATATATG
25	0171_F	CAATGGCAGTTATAAGAAGGCTCCC

26	0171_R	ATCCACCAAAGTTGTTTAAATCTTGTC
27	trxB1_F	CGGCAGTGGTCCAGCTGGATTGTC
28	trxB1_R	TAATTCATCACCACTTATATCATAAAAACC
29	spoIIID_F	GTAAGACAAACCGCCAAGAC
30	spoIIID_R	CCCCCTCTAATATGTCTCTCTG
31	prdF_F	GGAATAATCTTCATGGATGGTGG
32	prdF_R	CAACTGTAACGTCTCCTCTTATTATTCC
33	prdA_F	AGCTGTATTAGATATACAACCAATCGC
34	prdA_R	CCCCACATTATAGTAGTATTTAACTCACC
35	2078_F	TTAAAGGGACAGAAGTTCTTAAAGG
36	2078_R	GCTTTTTCTACATCTACTTTTACATCACC
37	atpG_F	CAAACAACACGTGGAATTAGAAATG
38	atpG_R	GTAATCACTGTTTCCTTTTTACCTTTC
39	3098_F	GACTTGGAAGATGGATTGAATC
40	3098_R	GTAATTACTAATCCATCTGCTCCC
41	1025_F	CAACAGGGACTTGTGCTACAG
42	1025_R	ATTCCTTTTAACTTTTGTATTACATCTTC
43	0571_F	GGAGCATATGTCTGGGAAGATG
44	0571_R	GATAGGGAGTCTAAAAAGTTCTTCTG
45	3099_F	GTAGTAGGAACTCCAGATGGAAATG
46	3099_R	CCTCAAATGTGTTAGCGTAGTCAAC
47	3100_F	GGCAGACTGGGAGAGGGTATC
48	3100_R	GAGTTCCATATCTCCCAAATACC
49	3388_F	GGGGAATGTCTGATGTTTCAAG
50	3388_R	GAATTAGCTCTCACTCTATCTGCCC
51	pabC_F	CAAGAGACGTATGAGAAGGGG
52	pabC_R	TTTAAAAATATACCATCATTATAACCAGTC
53	0758_F	GCTAAGGCAATTGCTATGGG
54	0758_R	AATCTTTTTCTCAATTCCTCATCC
55	0549_F	ATGAGTGCGTTGCTTGTGGTAG
56	0549_R	TCTCAATTACAGATGCTGGACATATC

57	rnhB_F	GAGAACGAAGGCTATGATAAAGG
58	rnhB_R	TTTGCCTCACTCAGTTTCTTAGAG
59	2586_F	TGGAGGAGCTAGAAATGGATTC
60	2586_R	GCTGCAACACCTGGAAGTCC
61	rbsR_F	GAAGGGGCCTATATAGCTACAAAAC
62	rbsR_R	TTCCTTCAAATACATAAGCAGAATTG
63	gatC_F	CACTTGCGGGATATGATGTAGC
64	gatC_R	AAAGTTCTCTGTCTCCATATTTTGC
65	2642_F	ACACACATGGTTATCTTCTAAGAGATG
66	2642_R	ATCTAAAATATATGATGGGTCAGCC
67	1455_F	GATGGGAAAAGATAATGTCTATAACTCC
68	1455_R	AATAGTTAATTCATAATTGCCTTCTCC
69	1395_F	TTACAGGAAACTTCTCTCTTGATGATAG
70	1395_R	GTGTATAAGAGCCAGCAATCAATAC
71	2269_F	GTGAGATAGGTGCATATGCAGCTC
72	2269_R	CCTCATCTCCTTCATTAGTATAGTTCC
73	licB_F	GAGGATGCAGCAAAGGAAAAC
74	licB_R	ACTGGAATATTATATGGTTTTGCC
75	1905_F	GTGATAGGAAGTAAATGGAGGGC
76	1905_R	GAAATTCTCTTAATAAGTCCATCTCTTTC
77	1449_F	GATGCGTTCGAATGGAGCTAC
78	1449_R	GAATTTGTTTACTGCTGCTACTGC
